# Case Report: Various Abnormalities in Lipid and Glucose Metabolism Induced by Capecitabine

**DOI:** 10.3389/fonc.2021.664475

**Published:** 2021-05-04

**Authors:** Takatoshi Anno, Tomoki Yamatsuji, Koichi Tomoda, Shuhei Nakanishi, Hideaki Kaneto

**Affiliations:** ^1^ Department of General Internal Medicine 1, Kawasaki Medical School, Okayama, Japan; ^2^ Department of Surgery, Kawasaki Medical School, Okayama, Japan; ^3^ Department of Diabetes, Endocrinology and Metabolism, Kawasaki Medical School, Kurashiki, Japan

**Keywords:** capecitabine, metabolic disorder, dyslipidemia, hypertriglyceridemia, hypercholesterolemia, hyperinsulinemia

## Abstract

Capecitabine has been used for the treatment of various types of tumors. The rare side effects induced by capecitabine have been reported as hypertriglyceridemia, acute pancreatitis associated with hypertriglyceridemia and hypertriglyceridemia complicated with hyperglycemia. The mechanisms of capecitabine-induced hypertriglyceridemia are unclear. In this report, we present a subject with sigmoid colon cancer and capecitabine-induced dyslipidemia. LDL-cholesterol level was markedly elevated throughout the long period of treatment with capecitabine. In addition, triglyceride level was high and not stable during the treatment period. Her dyslipidemia was ameliorated by the treatment with 5 mg of rosuvastatin, which is one of the HMG-CoA reductase inhibitors.

## Background

Capecitabine (XELODA^®^) has been used for the treatment of various types of tumors, such as breast, colorectal, gastric, head and neck carcinoma. Capecitabine is an oral fluoropyrimidine which is converted to 5-fluorouracil (5-FU) inside tumor cells *in vivo*, resulting in high drug concentrations. The rare side effects induced by capecitabine have been reported as follows: hypertriglyceridemia (1–9) and acute pancreatitis associated with hypertriglyceridemia (10–12), although the frequent adverse events of capecitabine are diarrhea, hand-foot syndrome, hyperbilirubinemia, and lymphopenia. The mechanisms of capecitabine-induced hypertriglyceridemia are unclear. Moreover, capecitabine-induced hypertriglyceridemia is sometimes complicated with hyperglycemia (13–15). In this report, we present a subject with sigmoid colon cancer and capecitabine-induced dyslipidemia. Low-density lipoprotein (LDL)-cholesterol level was markedly elevated throughout the long period of treatment with capecitabine. In addition, triglyceride level was high and not stable during the treatment period. Her dyslipidemia was ameliorated by the treatment with 5 mg of rosuvastatin, which is one of the 3-hydroxy-3-methylglutaryl coenzyme A (HMG-CoA) reductase inhibitors.

## Case presentation

A 52-year-old Japanese woman was referred and brought to an emergency room with symptoms of fever and abdominal pain. Her height, body weight and body mass index were 154.2 cm, 66.4 kg and 27.9 kg/m^2^, respectively. She had no remarkable past history and family history. Her vital signs were as follows: temperature, 37.7°C; blood pressure, 108/74 mmHg; heart rate, 108 beats/min; oxygen saturation, 95% (room air). **Table 1** shows laboratory data in an emergency room. Her infection markers were markedly elevated: white blood cell, 22850/μL (neutrophil, 93.6%); C-reactive protein, 17.38 mg/dL; procalcitonin, 69.58 ng/mL. Renal function was almost within the normal range, but liver dysfunction was observed: asparate aminotransferase (AST), 39 U/L; alanine transaminase (ALT), 31 U/L; alkaline phosphatase (ALP), 517 U/L; γ-glutamyl transpeptidase (γ-GTP), 117 U/L; lactate dehydrogenase (LDH), 276 U/L. In addition, her diabetes-associated data were as follows: plasma glucose, 419 mg/dL; hemoglobin A1c (HbA1c), 9.0%; plasma insulin 16.4 μU/mL. Lipid-associated data were almost within the normal range (total cholesterol, 129 mg/dL; Low Density Lipoprotein (LDL)-cholesterol, 89 mg/dL; High Density Lipoprotein (HDL)-cholesterol, 15 mg/dL; triglyceride, 102 mg/dL). Abdominal computed tomography (CT) and enhanced abdominal CT revealed a tumor in the sigmoid colon lesion, gastrointestinal perforation with free air, ascites and peritonitis. She immediately underwent emergency surgery and was diagnosed as sigmoid colon cancer, invasion to the abdominal wall and uterus and gastrointestinal perforation in the lesion of sigmoid colon cancer. Histopathological diagnosis was adenocarcinoma, and genomic analysis revealed the presence of activating mutations in the *KRAS* genes. Although during hospitalization period we treated her with intensive insulin therapy to obtain good glycemic control, she took 500 mg/day of metformin at discharge. At that time, her diabetes and dyslipidemia-associated data were as follows: plasma glucose, 145 mg/dL; HbA1c, 7.5%; total cholesterol, 256 mg/dL; LDL-cholesterol, 140 mg/dL; HDL-cholesterol, 28 mg/dL; triglyceride, 412 mg/dL. After emergency surgery, we started chemotherapy with 205 mg×1 day of oxaliplatin and 3,000 mg×14 days of capecitabine every 3 weeks. Since after 3 courses of this chemotherapy she suffered from bone marrow suppression, we decreased 80% dose of capecitabine as 2,400 mg×14 days and continued for more 3 courses of chemotherapy. Chemotherapy was effective for her tumor, and residual sigmoid colon cancer was not detected on positron emission tomography (PET)-CT. Therefore, she was once observed without chemotherapy including capecitabine.

**Table 1:** Laboratory data in an emergency room in this subject.

Variable	Result	Reference range	Variable	Result	Reference range
**Peripheral blood**			**Diabetes and Dyslipidemia marker**		
White blood cells (/μL)	22850	3300 – 8600	Plasma glucose (mg/dL)	419	
Neutrophil (%)	93.6	52.0 – 80.0	Hemoglobin A1c (%)	9.0	4.9 – 6.0
Red blood cells (×10^4^/μL)	330	386 – 492	Total cholesterol (mg/dL)	129	142 – 248
Hemoglobin (g/dL)	8.6	11.6 – 14.8	LDL cholesterol (mg/dL)	89	65 – 139
Hematocrit (%)	26.1	35.1 – 44.4	HDL cholesterol (mg/dL)	15	40 – 103
Platelets (×10^4^/μL)	62.6	15.8 – 34.8	Triglyceride (mg/dL)	102	30 – 149
**Blood biochemistry**			**Infectious marker**		
Total protein (g/dL)	6.8	6.6 – 8.1	CRP (mg/dL)	17.38	<0.14
Albumin (g/dL)	2.6	4.1 – 5.1	Procalcitonin (ng/mL)	69.58	0.00 – 0.05
Globulin (g/dL)	4.2	2.2 – 3.4	**Blood Gas Analysis**		
Total bilirubin (mg/dL)	0.7	0.4 – 1.5	pH	7.510	7.360 – 7.460
AST (U/L)	39	13 – 30	PCO_2_ (mmHg)	30.5	34.0 – 46.0
ALT (U/L)	31	7 – 23	PO_2_ (mmHg)	164.0	80.0 – 90.0
LDH (U/L)	276	124 – 222	$\text{HC}{\text{O}}_{3}^{-}(\text{mEq/L})$	24.1	24.0 – 32.0
ALP (U/L)	517	106 – 322	BE (mEq/L)	1.7	-2.5 – 2.5
γ-GTP (U/L)	117	9 – 32	SO_2_ (%)	99.2	95.0 – 98.0
BUN (mg/dL)	6	8 – 20	Lactate (mEq/L)	1.10	0.63 – 2.44
Creatinine (mg/dL)	0.56	0.46 – 0.79	**Coagulation fibrinolytic system-related antibodies**		
Cholinesterase (U/L)	245	201 – 421	PT-sec (sec)	15.5	9.3 – 12.5
Uric acid (mg/dL)	2.9	2.6 – 5.5	PT-INR	1.30	0.85 – 1.13
Creatine Kinase (U/L)	27	41 – 153	PT-activity (%)	58.4	80.7 – 125.2
Amylase (μg/dL)	25	44 – 132	APTT (sec)	35.3	26.9 – 38.1
Sodium (mmol/L)	131	138 – 145	D-dimer (μg/mL)	2.40	<1.0
Potassium (mmol/L)	3.6	3.6 – 4.8			
Chloride (mmol/L)	93	101 – 108			

AST, aspartate aminotransferase; ALT, alanine aminotransferase; LDH, lactate dehydrogenase; ALP, alkaline phosphatase; γ-GTP, γ-glutamyl transpeptidase; BUN, blood urea nitrogen; LDL, Low-density lipoprotein; HDL, High-density lipoprotein; CRP, C-reactive protein; BE, Base Excess; PT, prothrombin; PT-INR, PT-international normalized ratio; APTT, activated partial thromboplastin time.

After emergency surgery and through the period of chemotherapy, she was treated with 500 mg/day of metformin and her glycemic control was good (HbA1c: 6.2 -6.7%). However, her triglyceride level was elevated before the chemotherapy and was not stable during the period of chemotherapy. In addition, her LDL-cholesterol level was markedly elevated during the period of chemotherapy including capecitabine. We examined fasting data about dyslipidemia in more detail before starting course 6 of chemotherapy. Laboratory data about dyslipidemia were as follows (**Table 2**): total cholesterol, 312 mg/dL; LDL-cholesterol, 196 mg/dL; HDL-cholesterol, 46 mg/dL; triglyceride, 316 mg/dL; remnant-like particle (RLP)-cholesterol, 17.0 mg/dL; lipoprotein lipase (LPL), 95 ng/mL; apolipoprotein A-I, 126 mg/dl; apolipoprotein A-II, 26.1 mg/dl; apolipoprotein B, 181 mg/dl; apolipoprotein C-II, 9.8 mg/dl; apolipoprotein C-III, 23.7 mg/dl; apolipoprotein E, 10.7 mg/dl. In addition, lipoprotein fractions showed very low density lipoprotein (VLDL) and midband fractions were high (VLDL, 21%; midband, 39%). As shown in **Table 2**, LDL-cholesterol level during the period of chemotherapy (196-214 mg/dL) was markedly higher compared to before (80-140 mg/mL). LPL level was as low as 95 ng/mL (reference range 164 – 284 ng/mL) after course 5 of the chemotherapy including capecitabine. Fasting laboratory data about diabetes were as follows; plasma glucose, 128 mg/dL; plasma insulin, 27.5 μU/mL; HbA1c, 6.3%; glycoalbumin 15.1%. As shown in **Table 2**, plasma insulin level after starting the chemotherapy (27.5 μU/mL) was markedly higher compared to before (16.4 μU/mL).

**Table 2:** Time course of various dyslipidemia and diabetes markers before and after therapy.

Dyslipidemia marker	in an emergency room	1 month after admission	before starting course 2 of chemotherapy	before starting course 5 of chemotherapy	before starting course 6 of chemotherapy	1 month after course 6 of chemotherapy and 1 month after starting statin	3 months after course 6 of chemotherapy and 3 months after starting statin	Reference range
Total cholesterol (mg/dL)	129	256	333	377	312	215	186	142 - 248
LDL cholesterol (mg/dL)	89	140	162	214	196	123	107	65 – 139
HDL cholesterol (mg/dL)	15	28	42	49	46	47	44	40 – 90
Triglyceride (mg/dL)	102	421	632	574	316	297	163	40 – 149
RLP- cholesterol (mg/dL)	N/A	N/A	N/A	N/A	17.0	10.2	6.2	0.0 – 7.5
lipoprotein fractions								
VLDL (%)					21	21	13	3 - 19
Midband (%)					39	38	28	
LDL (%)					28	23	36	46 - 68
HDL (%)					12	18	23	22 - 74
Lipoprotein lipase (ng/mL)					95			164 - 284
Diabetes marker								
Plasma Glucose (mg/dL)	419	145	134	128	143	143	114	
Hemoglobin A1c (%)	9.0	7.5	6.7	6.3	6.3	6.5	6.6	4.9 – 6.0
Glycoalbumin (%)	27.5			15.1	17.2	15.7	15.5	12.4 – 16.3
Plasma insulin (μU/mL)	16.4			27.5	25.1	14.6	13.5	0.0 – 10.0

LDL, Low-density lipoproteins; HDL, High-density lipoprotein; RLP, remnant-like particle.

**Figure 1:**
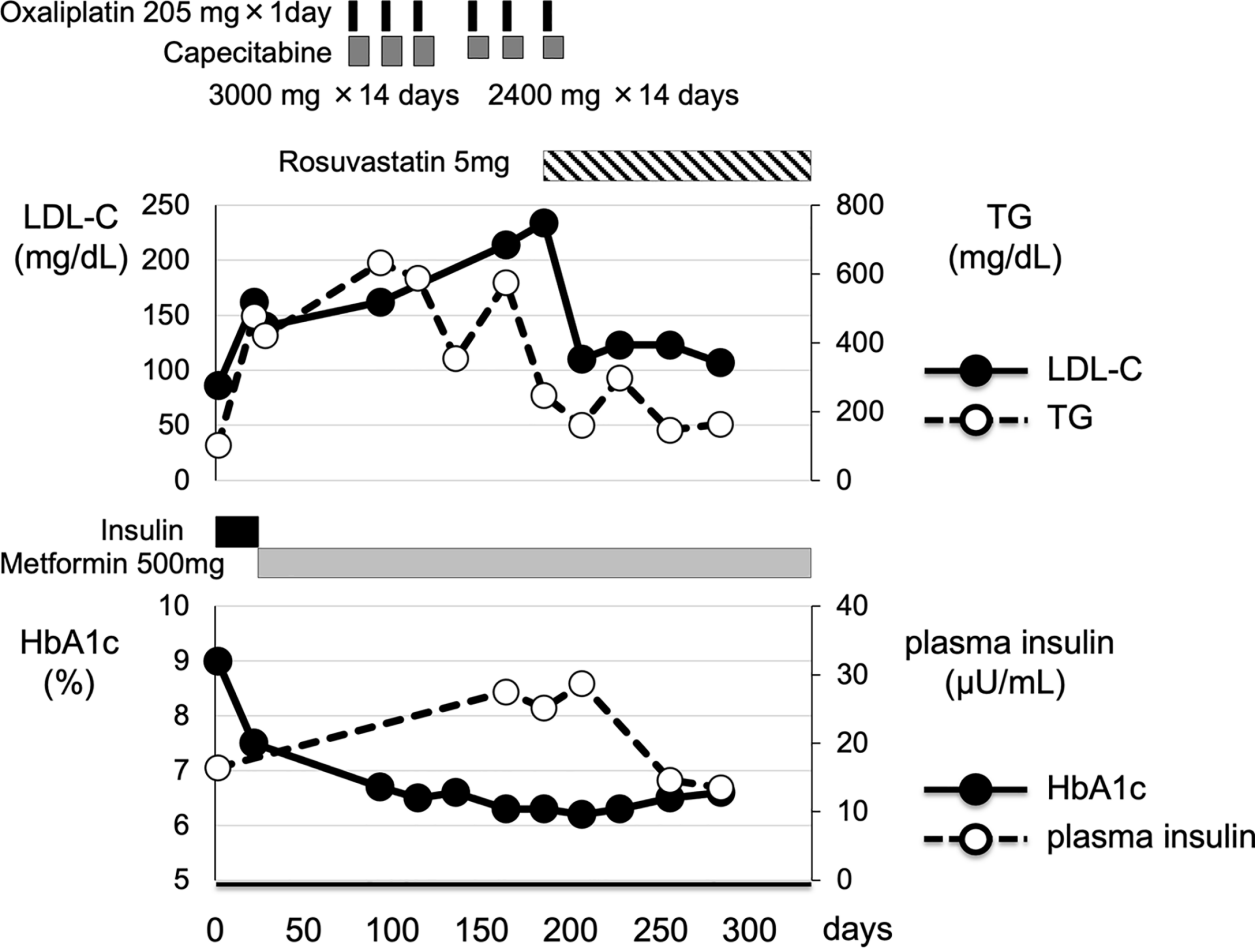
Clinical time course of clinical parameters in this subject. After emergency surgery, this patient underwent 6 courses of chemotherapy including capecitabine. After emergency surgery, she was treated with 500 mg of metformin for diabetes mellitus. In addition, through the chemotherapy including capecitabine, her plasma insulin level and LDL-cholesterol level were markedly elevated. And then, she was treated with 5 mg of rosuvastatin, one of the HMG-CoA reductases, for the treatment of dyslipidemia. Her plasma insulin and LDL-cholesterol level were markedly decreased.

After then, we started 5 mg/day of rosuvastatin for the treatment of dyslipidemia, and after then LDL-cholesterol level was decreased to 110 mg/dL and continued to be within normal range. VLDL and midband fractions on lipoprotein fractions were markedly decreased (VLDL, from 21% to 13%; midband, from 39% to 28%). Furthermore, as shown in **Figure 1**, her hyperinsulinemia was markedly improved (plasma insulin, 13.5 μU/mL) compared to that during the chemotherapy (25.1-27.5 μM/mL), although plasma insulin level was still slightly higher compared to its reference range presumably due to overweight.

## Discussion

Herein, we report a case of sigmoid colon cancer and capecitabine-induced dyslipidemia (increase of LDL-cholesterol, reduction of LPL activity) and metabolic disorder (hyperinsulinemia). It is known that hypertriglyceridemia is sometimes induced by capecitabine, although the mechanisms of capecitabine-induced hypertriglyceridemia are unclear. In this subject, however, LDL-cholesterol was markedly elevated through the treatment with capecitabine. Her dyslipidemia was well treated with an HMG-CoA reductase inhibitor throughout the period of the treatment with capecitabine and improved after the end of chemotherapy.

Our patient had hypertriglyceridemia and type 2 diabetes mellitus just after emergency surgery and before the chemotherapy including capecitabine. However, her plasma insulin level and LDL-cholesterol level were markedly elevated during the period of chemotherapy including capecitabine, although her glycemic control was good with the treatment of metformin therapy. Insulin resistance is caused by metabolic syndrome and various metabolic disorders especially in obese subjects. In addition, it is known that the decreased insulin effect, which is induced by insulin resistance, leads to the reduction of LPL activity (16). Her LPL level was as low as 95 ng/mL (reference range 164 – 284 ng/mL) after course 5 of the chemotherapy including capecitabine, and her insulin level was markedly elevated at that time, although her glycemic control continued to be good. In addition, she had both hypercholesterolemia and hypertriglyceridemia. Her triglyceride level was high before the chemotherapy, and LDL-cholesterol level was markedly elevated during the period of capecitabine therapy. Her dyslipidemia was well treated with an HMG-CoA reductase inhibitor and improved after the end of chemotherapy including capecitabine. Her RLP-cholesterol, which is known to stagnate the metabolism of triglyceride, was increased. RLP-cholesterol is elevated in patients with metabolic syndrome (17). And increased remnant lipoprotein leads to an abnormal concentration of triglyceride, and lipoprotein fractions show elevation of VLDL and midband (18). Her plasma insulin level was markedly elevated during the period of chemotherapy including capecitabine These data suggest that she was under metabolic disorder conditions during the period. In addition, her RLP-cholesterol level and VLDL and midband lipoprotein fractions were elevated presumably due to decreased LPL activity during the period of chemotherapy. In such conditions, the patient was exposed to hypercholesterolemia and hypertriglyceridemia probably together with insulin resistance. There were several reports showing capecitabine-induced hypertriglyceridemia, capecitabine-induced hypertriglyceridemia complicated with acute pancreatitis and capecitabine-induced hypertriglyceridemia complicated with hyperglycemia. We think that such reported side effect is one of a wide range of capecitabine-induced abnormalities in lipid and glucose metabolism in the whole body. The mechanism of capecitabine-induced various abnormalities in lipid and glucose metabolism remains unclear, although capecitabine-induced abnormalities are usually accompanied by mixed disturbance of the metabolic profiles. However, various abnormalities of our patient profile clearly indicated that capecitabine-induced metabolic disorder was associated with hyper insulinemia and insulin resistance together with increased triglyceride, LDL-cholesterol and RLP-cholesterol, increased VLDL and midband fractions, and increased blood glucose or glycated hemoglobin. Some reports suggest that the development of capecitabine-induced hypertriglyceridemia occurs more frequently in those with past and/or existing conditions associated with dyslipidemia, for example, obesity, hypertension, or diabetes mellitus. According to the U.S. Food & Drug Administration, the incidence of treatment-related grade 3-4 hypertriglyceridemia is 0.1–0.2% for capecitabine as monotherapy or as part of combination therapy. Although capecitabine-induced metabolic abnormality is a rare complication, it is possible that one of the mechanisms could be a combination of various metabolic abnormalities as observed in this patient.

There is a limitation in this study. Since we started rosuvastatin before starting course 6 of the chemotherapy, we think that the decrease of LDL-cholesterol was affected by statin therapy. We thought that it would be better not to start statin in order to clearly show the influence of capecitabine on lipid and glucose metabolism but that it would be quite problematic from the ethical point of view not to start any treatment for severe dyslipidemia. Moreover, capecitabine belongs to fluorinated pyrimidine derivatives, and it is possible that fluorinated pyrimidine derivatives increased oxidative stress, which led to metabolic abnormalities in this subject. If we had measured oxidative stress markers such as oxidized LDL, it would have helped us to strengthen such hypotheses.

Taken together, we should bear in mind that capecitabine therapy could bring out not only hypertriglyceridemia but also a wide range of abnormal lipid and glucose metabolism such as hyperinsulinemia, reduction of LPL activity and increase of LDL-cholesterol. In addition, we think that this case is very important because already reported capecitabine-induced hypertriglyceridemia could be one of a wide range of abnormalities in lipid and glucose metabolism accompanied by hyperinsulinemia, insulin resistance, decreased LPL activity and hypercholesterolemia. Therefore, we have to check various lipid and glucose metabolic disorders during the period of treatment with capecitabine.

## Data Availability Statement

The raw data supporting the conclusions of this article will be made available by the authors, without undue reservation.

## Ethics Statement

Written informed consent was obtained from the individual(s) for the publication of any potentially identifiable images or data included in this article.

## Author Contributions

TA researched data and wrote the manuscript. TY, KT, and SN researched data and contributed to the discussion. HK reviewed the manuscript. All authors contributed to the article and approved the submitted version.

## Conflict of Interest

The authors declare that the research was conducted in the absence of any commercial or financial relationships that could be construed as a potential conflict of interest.
